# Supplementary material for: Supplementing Low-Sodium Bicarbonate–Calcic (Lete)® Water: Effects in Women on Bone and Systemic Metabolism
Source: Metabolites. 2023 Oct 24;13(11):1109. doi: 10.3390/metabo13111109 (PMC10673306; doi:10.3390/metabo13111109)
Supplement: Supplementary file 1 [file metabolites-13-01109-s001.zip › metabolites-2642067-supplementary.pdf]

Supplementing low-sodium bicarbonate-calcic (Lete)® water: effects in women on bone and systemic metabolism.

Carmen Marino‡[1,2] , Imma Pagano‡[1,3], Giuseppe Castaldo[2], Manuela Grimaldi[1], Maria D'Elia[1,5], Angelo Santoro[1,4], Aurelio Conte[2,] Paola Molettieri[2], Chiara Parisella[2], Michela Buonocore[6], Anna Maria D'Ursi[1,5]*, Luca Rastrelli[1,5]*

‡ authors contributed equally to the work

1.Department of Pharmacy, University of Salerno, Via Giovanni Paolo II 132, 84084 Fisciano (SA), Italy. cmarino@unisa.it, asantoro@unisa.it ,magrimadi@unisa.it ,mdelia@unisa.it, rastrelli@unisa.it, dursi@unisa.it

2.Department of Pharmacy and Ph.D. Program in Drug Discovery and Development, University of Salerno, via Giovanni Paolo II, 132, 84084 Fisciano, Salerno, Italy.

3.NutriKeto_LAB Unisa - "San Giuseppe Moscati" National Hospital (AORN), Contrada Amoretta, 83100 Avellino (AV), Italy. giuseppecastaldo@yahoo.it, paolamolettieri@gmail.com

4.Department of Pharmacy, Scuola di Specializzazione in Farmacia Ospedaliera, University of Salerno, via Giovanni Paolo II,132, 84084, Italy.

5.NBFC, National Biodiversity Future Center, Palermo 90133, Italy.rastrelli@unisa.it, dursi@unisa.it

6.Department of Chemical Sciences, University of Naples Federico II, Complesso Universitario di Monte Sant'Angelo, Via Cinthia 21, 80126 Naples, Italy

* Correspondence:

Correspondence: Anna Maria D'Ursi and Luca Rastrelli, PhD, Department of Pharmacy, University of Salerno, Via Giovanni Paolo II, 132, 84084 Fisciano (SA), email: dursi@unisa.it; rastrelli@unisa.it; Tel.: 089969748

Supplementary Table S1. Eating habits at t0. Number of patients taking at least once a week a portion of the tabulated food.

Supplementary Table S2 Intervention group and control group's clinical average ± standard deviation parameters at t0. *P-value* was calculated using *T-test*.

Supplementary Table S3 Intervention group's clinical average ± standard deviation parameters at t0 and t6. *P-value* was calculated using *T-test*.

Supplementary Table S4 Control group's clinical average ± standard deviation parameters at t0 and t6. *P-value* was calculated using *T-test*.

Supplementary Table S5. Intervention group and control group's clinical average ± standard deviation parameters at t6. *P-value* was calculated using *T-test*.

Supplementary Table S6. Important serum and urine metabolites identified by Fold Change and logarithmic Fold Change (log2(FC)) parameters calculated.

Supplementary Table S7. Important serum and urinary metabolites identified by t-tests values, p-values (Threshold <0.05), logarithmic p-values and False Discovery Rate (FDR) parameters calculated for the most statistically significative compounds.

Supplementary Figure S1. Sample prediction area plot carried out using Centroid (a) Maximum distance (b) and Mahalanobis (c) showing the distribution of serum polar extract of intervention group at T0 (1) vs intervention group at T6 (2) in validation aerea

Supplementary Figure S2. Sample prediction area plot carried out using Centroid (a) Maximum distance (b) and Mahalanobis (c) showing the distribution of urine polar extract intervention group at T0 (1) vs intervention group at T6 (2) in validation aerea.

Supplementary Figure S3. Sample prediction area plot carried out using Centroid (a) Maximum distance (b) and Mahalanobis (c) showing the distribution of serum polar extract intervention group at T6 vs CTRL group at T6 in validation aerea.

Supplementary Figure S4. Sample prediction area plot carried out using Centroid (a) Maximum distance (b) and Mahalanobis (c) showing the distribution of urine polar extract intervention group at T6 vs CTRL group at T6 in validation aerea.

Supplementary Figure S5. PCA score plot for ^{1}H NMR data collected in 1D-NOESY spectra acquired at 600 MHz. Data represent the sera (a) and urine (b) from 79 women before (blue) and after (orange) bicarbonate-calcic water intake.

Supplementary Table S1. Eating habits at t0. Number of patients taking at least once a week a portion of the tabulated food.

**Food**	**YES**	**NO**
Wholemeal bread	121/121	0/121
Milk	119/121	2/121
Yoghurts	117/121	4/121
Cheese	121/121	0/121
Fish	100/121	21/121
Legume	110/121	11/121
Fruits	121/121	0/121
Vegetables	121/121	0/121
Sweets	80/121	41/121
Alcoholic drinks	61/121	60/121
Cereals	121/121	0/121
Pasta	121/121	0/121
Pizza	121/121	0/121
Meats	121/121	0/121

Supplementary Table S2 Intervention group and control group's clinical average ± standard deviation parameters at t0. *P-value* was calculated using *T-test*.

Parameters	Intervention-group-t0	CTRL-t0	p.value
ALB	4,12 ±1,13	4,23±1,01	0,65
ALP	66,55 ±20,78	60,54±16,22	0,16
Ca	9,37 ±0,38	9,41±0,38	0,67
U Ca	184,31 ±152,78	140,24±96,44	0,14
Ca^{++}	4,49 ±1,00	4,58±0,76	0,67
CL CR	103,79 ±56,39	91,86±33,58	0,27
COL	188,00 ±22,07	225,00±45,48	0,26
CREA	0,74 ±0,15	0,73±0,14	0,82
CREA U	1.078,45 ±539,81	925,11±306,83	0,13
GLIC	92,70 ±13,29	90,53±9,40	0,75
HCO^{3-}	29,89 ±2,55	30,40±2,80	0,40
HDL	56,00 ±13,11	63,00±14,17	0,53
INSUL	6,54 ±2,75	6,63±4,12	0,96
LDL	119,00 ±9,54	145,50±47,37	0,39
Mg	1,99 ±0,13	2,02±0,14	0,30
U Mg	78,67 ±49,03	68,11±30,43	0,26
Na	140,62 ±1,85	139,97±2,05	0,14
U Na	117,31 ±64,34	110,97±41,61	0,61
OSTEO	18,06 ±4,41	17,73±6,34	0,79
P	3,50 ±0,46	3,61±0,27	0,18
U P	667,33 ±417,94	525,38±259,89	0,08
SPEC. W	1.017,14 ±6,92	1.015,89±5,60	0,38
pH	5,76 ±0,74	5,84±0,67	0,61
PTH	69,29 ±17,97	60,76±21,18	0,06
VIT D	29,48 ±12,84	27,84±12,04	0,56

GLIC: glycemia (mg/ dL); CREA: creatinine (mg/dL); Na: sodium (mmol/L); Ca:calcium (mg/Dl); ALP: alkaline phosphatase (U/L); COL: cholesterol (mg/dL); HDL: Hight density lipoprotein (mg/ dL); LDL: Low density lipoprotein (mg/dL); P:phosphorus (mg/dL); Mg: magnesium (mg/dL); CREA U: urine creatinine (mg/24h); U Na: urinary sodium (mmol/24h); U Ca: urinary calcium (mmol/24h); U P: urinary phosphorus (mmol/24h); U Mg: urinary magnesium (mmol/24h); CL CR: creatinine clearance (ml/min); ALB: albumine (gr/dl); PTH: Parathormone (pg/ml); OSTEO: osteocalcine (mg/mL); 25-OHD: vitamine D (ng/ mL); INSUL: insulinemia (mcUI/ml); Ph; P.SPEC: specific weight; HCO^{3-}: bicarbonate (mmol/L); Ca^{++}: ionized calcium (mg/ dL).

Supplementary Table S3 Intervention group's clinical average ± standard deviation parameters at t0 and t6. *P-value* was calculated using *T-test*.

Parameters	Intervention-group-t0	Intervention_group_t6	p.value
ALB	4,12 ±1,13	4,4 ±0,1	1,00
ALP	66,55 ±20,78	66,9 ±19,16	0,85
Ca	9,37 ±0,38	9,6 ± 0,37	0,0007
U Ca	184,31 ±152,78	206,4 ±127,03	0,03
Ca^{++}	4,49 ±1,00	4,7 ±0,14	0,26
CL CR	103,79 ±56,39	111,6 ±51,68	0,46
COL	188,00 ±22,07	209,6 ±33,00	0,51
CREA	0,74 ±0,15	0,7 ± 0,09	0,90
CREA U	1.078,45 ±539,81	1.163,9 ±592,54	0,39
GLIC	92,70 ±13,29	83,38 ±6,25	0,13
HCO^{3-}	29,89 ±2,55	31,4 ±2,70	0,00
HDL	56,00 ±13,11	67,4 ± 14,25	0,02
INSUL	6,54 ±2,75	5,5 ± 2,68	0,95
LDL	119,00 ±9,54	139,7 ±29,88	0,52
Mg	1,99 ±0,13	2,0 ± 0,16	0,12
U Mg	78,67 ±49,03	91,5 ± 45,81	0,18
Na	140,62 ±1,85	140,1 ±2,22	0,10
U Na	117,31 ±64,34	150,0 ±62,66	0,01
OSTEO	18,06 ±4,41	16,8 ±5,28	0,02
P	3,50 ±0,46	3,6 ± 0,37	0,23
U P	667,33 ±417,94	667,3 ±417,94	1,00
SPEC. W	1.017,14 ±6,92	1.014,9 ± 5,64	0,04

pH	5,76 ±0,74	5,8 ± 0,62	1,00
PTH	69,29 ±17,97	71,2 ±19,91	0,63
VIT D	29,48 ±12,84	33,8 ± 13,55	0,02

GLIC: glycemia (mg/ dL); CREA: creatinine (mg/dL); Na: sodium (mmol/L); Ca:calcium (mg/Dl); ALP: alkaline phosphatase (U/L); COL: cholesterol (mg/dL); HDL: Hight density lipoprotein (mg/ dL); LDL: Low density lipoprotein (mg/dL); P:phosphorus (mg/dL); Mg: magnesium (mg/dL); CREA U: urine creatinine (mg/24h); U Na: urinary sodium (mmol/24h); U Ca: urinary calcium (mmol/24h); U P: urinary phosphorus (mmol/24h); U Mg: urinary magnesium (mmol/24h); CL CR: creatinine clearance (ml/min); ALB: albumine (gr/dl); PTH: Parathormone (pg/ml); OSTEO: osteocalcine (mg/mL); 25-OHD: vitamine D (ng/ mL); INSUL: insulinemia (mcUI/ml); Ph; P.SPEC: specific weight; HCO^{3-}: bicarbonate (mmol/L); Ca^{++}: ionized calcium (mg/ dL).

Supplementary Table S4 Control group's clinical average ± standard deviation parameters at t0 and t6. *P-value* was calculated using *T-test*.

Parameters	CTRL-t0	CTRL_t6	p.value
ALB	4,23±1,01	89.21±11,03	0,90
ALP	60,54±16,22	0,70±0,12	0,92
Ca	9,41±0,38	140,13±1,75	1,00
U Ca	140,24±96,44	9,41±1,36	0,74
Ca^{++}	4,58±0,76	60,19±15,29	0,32
CL CR	91,86±33,58	210,43±34,63	0,40
COL	225,00±45,48	65,59±14,59	0,44
CREA	0,73±0,14	140,18±33,21	0,43
CREA U	925,11±306,83	3,7±0,49	0,73
GLIC	90,53±9,40	2,02±0,13	0,20
HCO^{3-}	30,40±2,80	949,32±286,47	0,08
HDL	63,00±14,17	120,37±46,25	0,74
INSUL	6,63±4,12	147,78±99,30	0,83
LDL	145,50±47,37	525,37±259,89	0,77
Mg	2,02±0,14	66,32±27,82	1,00
U Mg	68,11±30,43	98,21±31,11	0,79
Na	139,97±2,05	4,18±0,24	0,72
U Na	110,97±41,61	55,21±13,25	0,36
OSTEO	17,73±6,34	19,95±8,63	0,21
P	3,61±0,27	31,64±13,017	0,36
U P	525,38±259,89	6,36±3,31	1,00
SPEC. W	1.015,89±5,60	5,85±0,67	0,35
pH	5,84±0,67	1014,70±5,36	0,93
PTH	60,76±21,18	30,52±3,83	0,18
VIT D	27,84±12,04	4,41±0,70	0,20

GLIC: glycemia (mg/ dL); CREA: creatinine (mg/dL); Na: sodium (mmol/L); Ca:calcium (mg/Dl); ALP: alkaline phosphatase (U/L); COL: cholesterol (mg/dL); HDL: Hight density lipoprotein (mg/ dL); LDL: Low density lipoprotein (mg/dL); P:phosphorus (mg/dL); Mg: magnesium (mg/dL); CREA U: urine creatinine (mg/24h); U Na: urinary sodium (mmol/24h); U Ca: urinary calcium (mmol/24h); U P: urinary phosphorus (mmol/24h); U Mg: urinary magnesium (mmol/24h); CL CR: creatinine clearance (ml/min); ALB: albumine (gr/dl); PTH: Parathormone (pg/ml); OSTEO: osteocalcine (mg/mL); 25-OHD: vitamine D (ng/ mL); INSUL: insulinemia (mcUI/ml); Ph; P.SPEC: specific weight; HCO^{3-}: bicarbonate (mmol/L); Ca^{++}: ionized calcium (mg/ dL).

Supplementary Table S5. Intervention group and control group's clinical average ± standard deviation parameters at t6. *P-value* was calculated using *T-test*.

Parameters	Intervention_group_t6	CTRL_t6	p-value
ALB	83,38 ±6,25	89.21±11,03	0,003
ALP	0,7 ± 0,09	0,70±0,12	0,11
Ca	140,1 ±2,22	140,13±1,75	0,48
U Ca	9,6 ± 0,37	9,41±1,36	0,15
Ca^{++}	66,9 ±19,16	60,19±15,29	0,04
CL CR	209,6 ±33,00	210,43±34,63	0,45
COL	67,4 ± 14,25	65,59±14,59	0,28
CREA	139,7 ±29,88	140,18±33,21	0,47
CREA U	3,6 ± 0,37	3,7±0,49	0,11
GLIC	2,0 ± 0,16	2,02±0,13	0,49
HCO^{3-}	1.163,9 ±592,54	949,32±286,47	0,02
HDL	150,0 ±62,66	120,37±46,25	0,010
INSUL	206,4 ±127,03	147,78±99,30	0,013
LDL	667,3 ±417,94	525,37±259,89	0,03
Mg	91,5 ± 45,81	66,32±27,82	0,002
U Mg	111,6 ±51,68	98,21±31,11	0,08
Na	4,4 ±0,1	4,18±0,24	0,04
U Na	71,2 ±19,91	55,21±13,25	4,44E-05
OSTEO	16,8 ±5,28	19,95±8,63	0,02
P	33,8 ± 13,55	31,64±13,017	0,28
U P	5,5 ± 2,68	6,36±3,31	0,09
SPEC. W	5,8 ± 0,62	5,85±0,67	0,24
pH	1.014,9 ± 5,64	1014,70±5,36	0,42
PTH	31,4 ±2,70	30,52±3,83	0,11
VIT D	4,7 ±0,14	4,41±0,70	0,011

GLIC: glycemia (mg/ dL); CREA: creatinine (mg/dL); Na: sodium (mmol/L); Ca:calcium (mg/Dl); ALP: alkaline phosphatase (U/L); COL: cholesterol (mg/dL); HDL: Hight density lipoprotein (mg/ dL); LDL: Low density lipoprotein (mg/dL); P:phosphorus (mg/dL); Mg: magnesium (mg/dL); CREA U: urine creatinine (mg/24h); U Na: urinary sodium (mmol/24h); U Ca: urinary calcium (mmol/24h); U P: urinary phosphorus (mmol/24h); U Mg: urinary magnesium (mmol/24h); CL CR: creatinine clearance (ml/min); ALB: albumine (gr/dl); PTH: Parathormone (pg/ml); OSTEO: osteocalcine (mg/mL); 25-OHD: vitamine D (ng/ mL); INSUL: insulinemia (mcUI/ml); Ph; P.SPEC: specific weight; HCO^{3-}: bicarbonate (mmol/L); Ca^{++}: ionized calcium (mg/ dL).

Supplementary Table S6. Important serum and urine metabolites identified by Fold Change and logarithmic Fold Change (log2(FC)) parameters calculated.

**Serum**	Fold Change	log2(FC)
Hypoxanthine	12.267	36.167
Ethanol	0.14964	-27.405
L-Arginine	0.17354	-25.267
L-Histidine	0.18232	-24.555
Pyruvic acid	0.2385	-2.068
3-Hydroxybutyric acid	0.28649	-18.034
Citric acid	0.28736	-17.991
Acetone	0.2923	-17.745
Isoleucine	0.30153	-17.296
Glycerol	0.33357	-1.584
Acetic acid	0.37096	-14.307
Methanol	0.42366	-1.239
Succinate	0.42765	-12.255
Betaine	22.077	11.426
L-Asparagine	21.978	11.361
Carnitine	20.423	10.302

Urine	Fold Change	log2(FC)
Fructose	0.031436	-49.914
Trimethylamine	53.005	24.061
L-Ornithine	42.797	20.975
Ascorbic acid	41.349	20.478
Myo-inositol	41.141	20.406
L-Fucose	0.25179	-19.897
D-Galactose	37.351	19.012
Glycine	32.604	1.705
L-Leucine	0.30695	-17.039
Pyruvic acid	3.138	16.498
Methionine	28.072	14.891
Caffeine	27.285	14.481
Gluconic acid	26.597	14.113
L-Histidine	26.465	14.041
Oxalacetic acid	24.951	13.191
Carnitine	23.441	12.291
L-Glutamine	22.827	11.908
Guanidoacetate	21.915	11.319
Acetic acid	21.853	11.278

L-Arginine	0.47252	-10.816
Sarcosine	20.839	10.593
Mannose	0.49226	-10.225
3-Hydroxyisobutyrate	20.198	10.142
3-Hydroxybutyric acid	20.189	10.136
L-Cysteine	20.121	10.087
L-Lactic acid	20.027	10.019

Supplementary Table S7. Important serum and urinary metabolites identified by t-tests values, p-values (Threshold <0.05), logarithmic p-values and False Discovery Rate (FDR) parameters calculated for the most statistically significative compounds.

**Serum**	t.stat	p.value	FDR
3-Hydroxybutyric acid	-14.12	1,09E-25	4,69E-24
L-Arginine	-88.468	1,84E-11	3,95E-10
Succinate	-77.884	8,86E-09	1,24E-07
Citric acid	-77.424	1,15E-08	1,24E-07
Betaine	71.843	2,61E-07	2,24E-06
Isoleucine	-69.839	7,80E-08	5,59E-06
D-Glucose	64.683	1,21E-05	7,45E-05
Hypoxanthine	55.886	9,97E-04	5,36E-03
L-Ornithine	-51.454	7,92E-03	3,66E-02
L-Lysine	51.298	8,50E-03	3,66E-02
Valine	50.543	1,20E-02	4,68E-02
Pyruvic acid	-46.155	8,14E-02	2,92E-01
Glycerol	-44.267	1.79e-05	5,92E-01
L-Leucine	-44.072	1,94E-01	5,96E-01
Carnitine	4.384	2,13E-01	6,11E-01
L-Phenylalanine	43.667	2,29E-01	6,15E-01

Urea	37.502	0.00024855	0.00060451
L-Histidine	-37.453	0.00025305	0.00060451
L-Threonine	34.831	0.00064327	0.0014558
Tyrosine	33.805	0.00091445	0.0019661
Glycine	31.814	0.0017686	0.0036214
Choline	30.001	0.0031428	0.0061428
Propylene glycol	2.935	0.0038398	0.0071787
L-Alanine	27.362	0.0069368	0.012378
L-Lactic acid	27.235	0.0071964	0.012378
Acetoacetate	26.576	0.00869	0.014372
2-Hydroxybutyric acid	2.607	0.010019	0.015956
Ethanol	-2.463	0.014863	0.022826
L-Glutamic acid	23.557	0.019729	0.029254
L-Asparagine	22.361	0.026764	0.038362
**Urine**	t.stat	p.value	FDR
Fructose	-99.992	2,96E-14	2.43e-16
L-Arginine	-90.193	9,92E-12	4,07E-10
Glycine	85.983	1,15E-10	3,14E-09
Trimethylamine	7.942	4,88E-09	1,00E-07
Mannose	-76.781	2,14E-08	3,24E-07

Myo-inositol	76.593	2,37E-08	3,24E-07
L-Leucine	-6.787	2,67E-06	3,13E-05
Alpha-Lactose	-6.649	5,50E-06	5,64E-05
L-Fucose	-65.882	7,55E-06	6,88E-05
Pyruvic acid	61.693	6,40E-05	5,25E-04
Hydroxyphenyllactic acid	-54.703	1,90E-03	1,42E-02
Hypoxanthine	50.419	1,35E-02	9,20E-03
L-Serine	-50.011	1,61E-02	1,02E-01
5-Aminopentanoate	44.165	1,94E-01	0.00011371
Creatinine	-4.324	2,82E-02	0.00015432
2-Oxopentanedioate	-42.923	3,21E-01	0.00016429
2-Hydroxybutyric acid	-41.726	5,15E-01	0.0002408
Dimethylamine	41.659	5,29E-01	0.0002408
Valine	-4.152	5,58E-01	0.00024084
L-Cysteine	38.946	0.00014923	0.00061183
Isoleucine	-38.716	0.00016257	0.00063033
L-Glutamine	38.504	0.00017594	0.00063033
2-Hydroxyisovalerate	-3.849	0.0001768	0.00063033
3-Hydroxybutyric acid	3.685	0.00032158	0.0010987
Caffeine	36.636	0.00034723	0.0011389

L-Cystine	36.199	0.00040567	0.0012794
Creatine	-35.276	0.00056088	0.0017034
L-Proline	34.581	0.00071305	0.0020882
L-Threonine	33.843	0.00091641	0.0025912
Ascorbic acid	33.682	0.00096758	0.0026447
L-Ornithine	32.845	0.0012788	0.0033827
L-Phenylalanine	31.895	0.0017445	0.0044702
Oxalacetic acid	30.331	0.0028658	0.007121
3-Hydroxyisobutyrate	-2.954	0.0036573	0.0088206
L-Alanine	29.099	0.0041816	0.0097968
Acetone	-26.923	0.0079259	0.018053
Isovaleric acid	25.347	0.012307	0.026584
Phenylacetate	25.344	0.012319	0.026584
Hippuric acid	24.402	0.015877	0.033382
D-Galactose	23.602	0.019589	0.040157

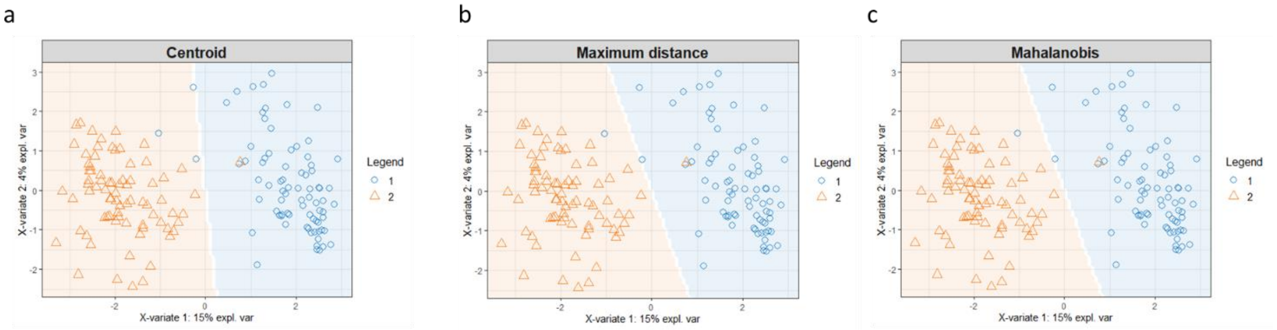

Supplementary Figure S1. Sample prediction area plot carried out using Centroid (a) Maximum distance (b) and Mahalanobis (c) showing the distribution of serum polar extract of intervention group at T0 (1) vs intervention group at T6 (2) in validation aerea

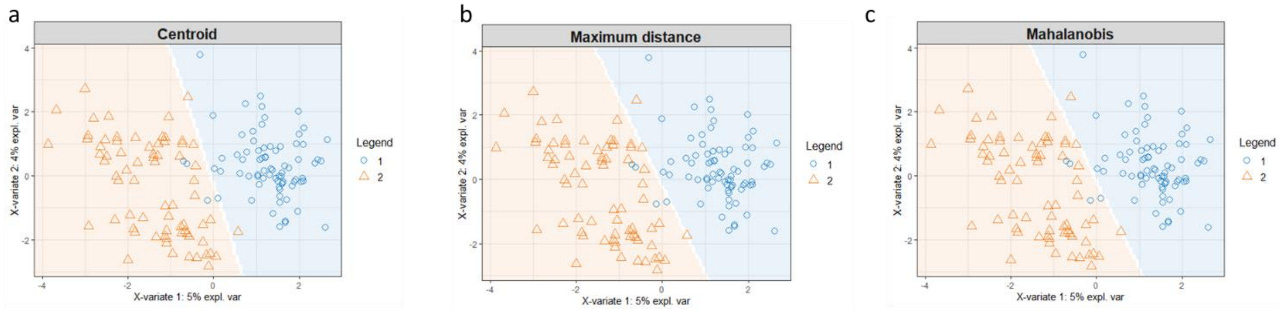

Supplementary Figure S2. Sample prediction area plot carried out using Centroid (a) Maximum distance (b) and Mahalanobis (c) showing the distribution of urine polar extract intervention group at T0 (1) vs intervention group at T6 (2) in validation aerea.

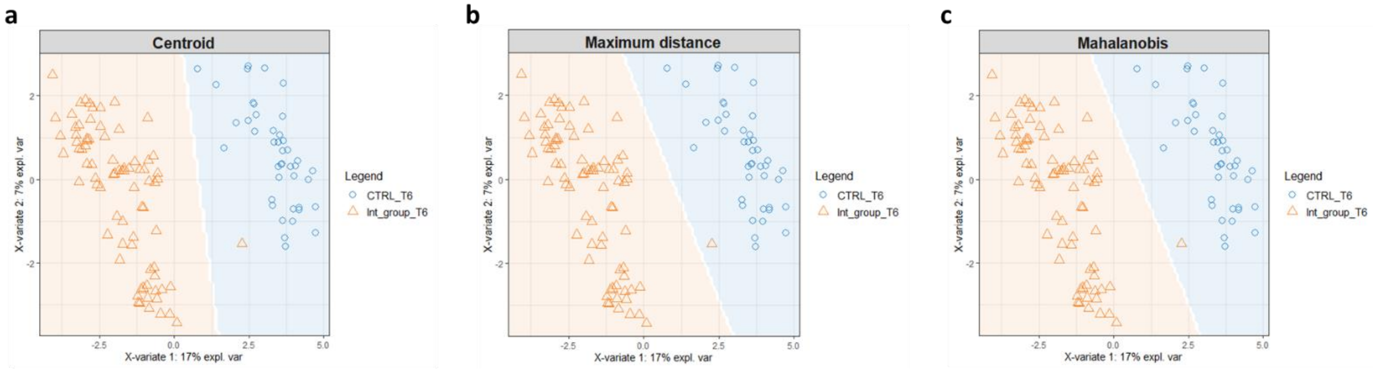

Supplementary Figure S3. Sample prediction area plot carried out using Centroid (a) Maximum distance (b) and Mahalanobis (c) showing the distribution of serum polar extract intervention group at T6 vs CTRL group at T6 in validation aerea.

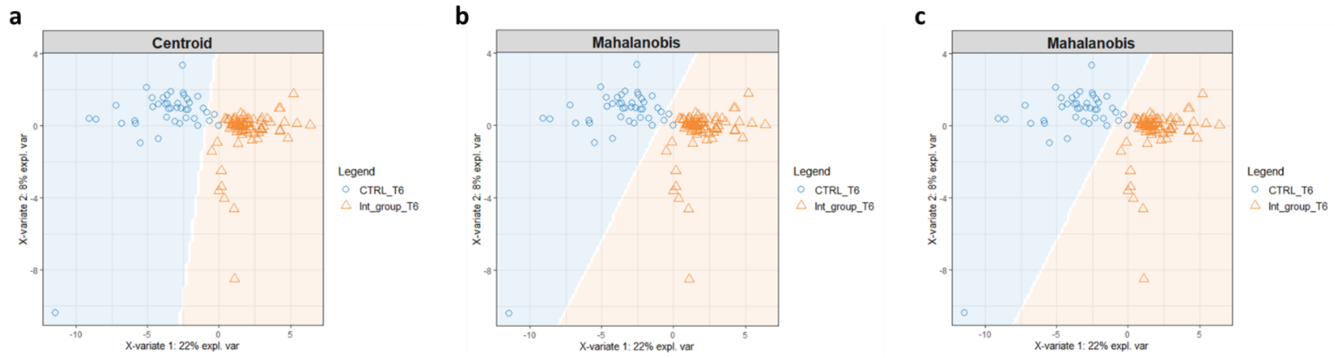

Supplementary Figure S4. Sample prediction area plot carried out using Centroid (a) Maximum distance (b) and Mahalanobis (c) showing the distribution of urine polar extract intervention group at T6 vs CTRL group at T6 in validation aerea.

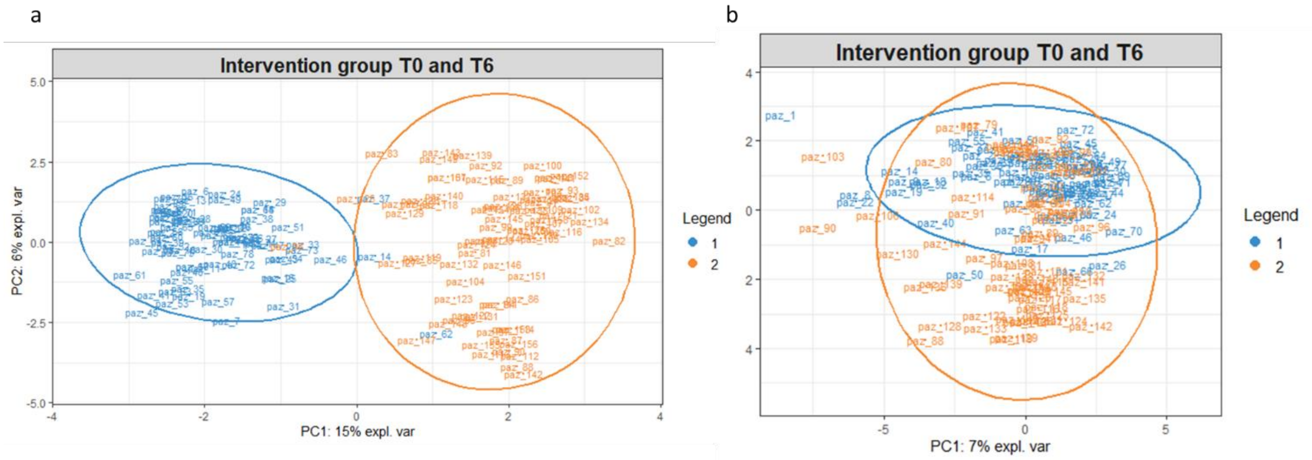

Supplementary Figure S5. PCA score plot for ^{1}H NMR data collected in 1D-NOESY spectra acquired at 600 MHz. Data represent the sera (a) and urine (b) from 79 women before (blue) and after (orange) bicarbonate-calcic water intake.
